# Prediction Error Stabilization and Long-Term Standard Results with a Monofocal Intraocular Lens

**DOI:** 10.3390/vision6010005

**Published:** 2022-01-13

**Authors:** Beatríz Macías-Murelaga, Gonzaga Garay-Aramburu, Roberto Bergado-Mijangos, Daniel Coello-Ojeda, Itziar Ozaeta, Pio Jésus Garcia-Gómez, Jesús Garrido-Fierro, Manuel Rodríguez-Vallejo, Joaquín Fernández

**Affiliations:** 1 Department of Ophthalmology, Cruces University Hospital, 48903 Barakaldo, Spain; beatrizdelierni.maciasmurelaga@osakidetza.eus; 2 Begiker-Ophthalmology Research Group, Department of Ophthalmology, Biocruces Bizkaia Health Research Institute, OSI Bilbao Basurto, Facultad de Medicina, Campus de Vitoria-Gasteiz, University of the Basque Country, UPV/EHU, Avenida Montevideo 18, 48013 Bilbao, Spain; gonzagaray@gmail.com; 3 Department of Ophthalmology, Araba University Hospital, 01004 Vitoria-Gasteiz, Spain; roberto.bergadomijangos@osakidetza.eus (R.B.-M.); danielantonio.coelloojeda@osakidetza.eus (D.C.-O.); iciar.ozaetaortizdeurbina@osakidetza.eus (I.O.); jesusmaria.garridofierro@osakidetza.eus (J.G.-F.); 4 Department of Ophthalmology, Quironsalud Hospital, 01002 Vitoria-Gasteiz, Spain; piojesus.garcia@quironsalud.es; 5 Qvision, Department of Ophthalmology, VITHAS Hospital, 04120 Almeria, Spain; joaquinfernandezoft@qvision.es

**Keywords:** intraocular lens, prediction error, stabilization

## Abstract

The aim of this study was to assess the stability and differences between objective (O-Rx) and subjective (S-Rx) refraction for the assessment of the prediction error (PE). A secondary aim was to report the results of a monofocal intraocular lens (IOL). 100 subjects were included for whom S-Rx and O-Rx were obtained for all visits, and for visual performance, posterior capsular opacification incidence and Nd:YAG rates at 12 months. Either S-Rx and O-Rx showed a hyperopic shift from 1 to 6 months (*p* < 0.05) and stabilization after 6 months. S-Rx was related with the axial length (rho = −0.29, *p* = 0.007), obtaining a major tendency towards hyperopia in short eyes implanted with high-power IOLs. O-Rx showed a myopic shift in comparison to S-Rx (*p* < 0.05). This resulted in a decrease of the number of eyes in ±0.50 D and ±1.00 D from 79 to 67% and from 94 to 90%, respectively. The median (interquartile range) uncorrected and corrected visual acuities were 0.1 (0.29) and 0 (0.12) logMAR, respectively, and seven eyes required Nd:YAG capsulotomy at 12 months. Some caution should be taken in PE studies in which O-Rx is used or S-Rx is measured in a 1-month follow-up. Constant optimization should be conducted for this IOL after S-Rx stabilization.

## 1. Introduction

Satisfaction after cataract surgery with the implantation of a monofocal intraocular lens (IOL) results from a multifactorial approach [1,2,3], which includes achieving the lowest possible postoperative residual refraction that allows distance spectacle independence [4]. Several formulas have been developed and compared through the prediction error (PE), which is the difference between the postoperative spherical equivalent (SE) obtained from the subjective refraction and the predicted refraction (PR) provided by the formula for a particular implanted IOL power [5]. Fortunately, standards for reporting results with monofocal IOLs and for formulae comparison have been developed to reduce the bias among results reported by several authors [6,7,8,9,10]. However, some studies still fail in reporting the formula used for selecting the implanted IOL power [11], use objective refraction for assessing the PE [12,13,14], and there is still a lack of consensus on refraction stabilization at one month [7] or longer periods of time [6]. The main aim of this study was to assess how PE could be influenced by a refraction procedure, objective versus subjective, at several periods of follow-up. A secondary aim was to assess the standard results obtained after 12 months of implantation for a monofocal preloaded hydrophobic IOL.

## 2. Materials and Methods

### 2.1. Subjects and Procedures

The study was approved by the Drug Research Ethics Committee of Euskadi (PS2018031) and was performed in adherence to the tenets of the Declaration of Helsinki. All patients provided informed consent to participate in the study.

100 subjects consecutively operated for cataract surgery between March 2019 and December 2019 at Araba University Hospital were prospectively followed during a 12-month period. The inclusion criteria were patients with cataract and preoperative monocular corrected distance visual acuity (CDVA) >0.1 logMAR with or without corneal astigmatism, following the standards of clinical practice of the public health site on which the study was conducted. Exclusion criteria included any active ocular diseases, chronic uveitis, retinal degenerations, glaucoma/ocular hypertension >24 mmHg, pseudoexfoliation syndrome, keratoconus, corneal endothelial dystrophies, previous intraocular surgery, clinical history of corneal laser refractive surgery, corneal ectasia, trauma, opacifications and the refusal to sign the informed consent.

Objective (TRK-2P, Topcon Corporation, Tokyo, Japan) and subjective refractions were obtained at all the follow-up visits by the same optometrist using the phoropter and an optotype chart located at 4 m; no corrections of the spherical refraction to 6 m or infinity were applied as a single formula for the IOL power calculation used in this study. Changes in corneal power and astigmatism between the preoperative and the 12-month visit were measured with the Sirius corneal topographer (Schwind eye-tech-solutions GmbH & Co. KG; Kleinostheim, Germany). The uncorrected distance visual acuity (UDVA) was measured at 4 m, and the CDVA with the subjective refraction was obtained at this distance without vergence correction. The contrast sensitivity function (CSF) was measured monocularly with a best distance correction at 4 m with the CC-100XP device (Topcon Corporation, Tokyo, Japan). The posterior capsular opacification and Nd-YAG rates were collected as dichotomous variables.

### 2.2. Intraocular Lens and Surgery

All patients were implanted with an aspherical non-toric hydrophobic monofocal preloaded IOL (AIALA DRY, LLASHP60-PL, AJL Ophthalmic SA; Alava, Spain), which is made of hydrophobic material with a c-loop platform with an optical diameter of 6 mm and total length of 13 mm. The IOL power was calculated using the constant recommended by the manufacturer (A = 119.7) and the Barrett formula integrated in the Lenstar LS 900 biometer (Haag-Streit AG, Koeniz, Switzerland). All surgeries were conducted by different surgeons with phacoemulsification through a 2.2 mm incision size at temporal location and while conducting a continuous circular capsulorhexis. The lens was removed by phacoemulsification using the surgeon’s preferred methodology and while implanting the preloaded monofocal IOL. The recorded intraoperative information was: presence of pupillary problems and any complications that occurred, including floppy iris syndrome (IFIS), posterior capsule tears, zonular dehiscence, vitreous prolapse or lens implantation difficulties, among others.

### 2.3. Statistical Analysis

The descriptive statistics are detailed in the results section as the mean ± standard deviation for normally distributed variables and as the median (interquartile-range) for non-normally distributed ones. The distributions were tested with the Kolmogorov–Smirnov test and through a histogram inspection. The one-sample Wilcoxon signed rank test was used for testing the null hypothesis of the median difference of PE equal to zero in order to assess if constant optimization was required. The median absolute error (AE) was also calculated as the absolute value of the PE. The stability of subjective and objective refractions was assessed with a Friedman test with a post-hoc paired comparison and Bonferroni correction. Correlations between the subjective refraction stability and axial length or IOL power were assessed with the Spearman rho. The assessment of confounding factors as corneal power changes between 1 and 12 months was evaluated with the paired *t*-test. The influence of biometric parameters and PE was descriptively evaluated with plots previously described in the literature [15]. The association between PCO or Nd:YAG and PE changes was evaluated with the Fisher’s exact test. SPSS version 24 software (SPSS Inc, Chicago, IL, USA) was used for the statistical analysis. The Refractive Analysis toolbox (v1.0.5) for MATLAB (R2009; MathWorks, Natick, MA, USA) was used to conduct the standard plots [16].

## 3. Results

### 3.1. Prediction Error

Fifty men and 50 women were included in the study. The demographic characteristics of the sample are shown in Table 1. 97% of the eyes achieved a CDVA ≤ 0.2 logMAR at the 12-month follow-up. The median PEs at one, six and 12 months were 0.20 (0.38), 0.32 (0.57) and 0.33 (0.64) D, respectively, and were significantly different from 0 for the three follow-up periods (*p* < 0.05), suggesting that constant optimization was required for each one of the visits. On the other hand, the median AEs at one, six and 12 months were 0.33 (0.33), 0.41 (0.55) and 0.43 (0.54) D. The percentage of eyes in ± 0.50 decreased from 79% at one month to 68% and 67% at six and 12 months, respectively. The percentage of eyes in ± 1.00 D decreased from 94% at one month to 91% and 90% at six and 12 months, respectively.

**Figure 1:**
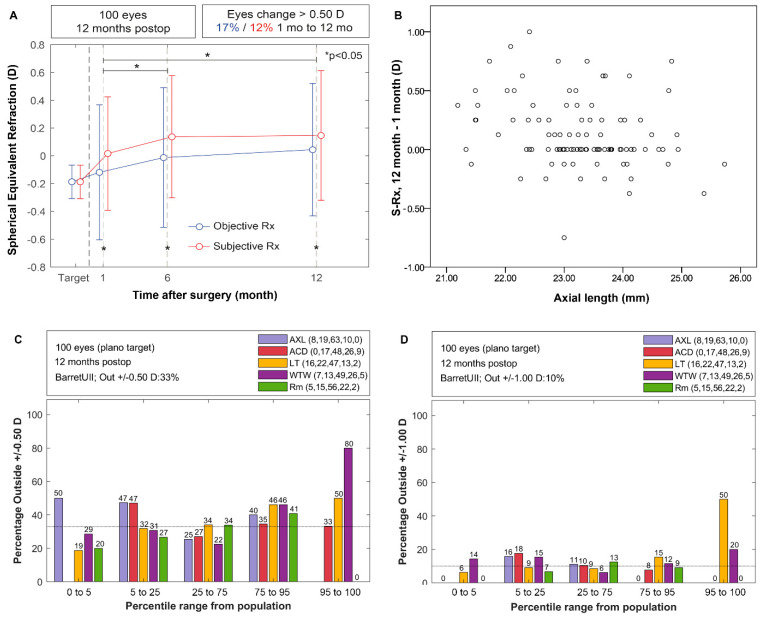
(**A**) Stability of spherical equivalent refraction; (**B**) Relationship between axial length and variation in spherical equivalent; (**C**) Assessment of % of eyes outside ±0.50 D and (**D**) outside ±1.00 D through percentiles defined from the healthy cataract population.

A statistically significant hyperopic shift was found between one month and six months, either for objective or subjective refraction; however, the refractive error was stable between six and 12 months (Figure 1A). The O-Rx was more myopic than the S-Rx for all the postoperative visits. The S-Rx was stable in 39% of eyes, 44% showed a hyperopic tendency and 17% a myopic tendency. Significant correlations were found between the difference in S-Rx (12–1 months) and IOL power (rho = 0.21, *p* = 0.03) or AXL (rho = −0.29, *p* = 0.007) (Figure 1B). The mean corneal power at 12 months was 43.88 ± 1.37 D, very close to the reported preoperative one of 43.91± 1.38 D (t = −0.74, *p* = 0.46).

Figure 1C,D shows the percentage of eyes outside +0.50 D and +1.00 D, respectively, stratified according to the percentiles of distribution of biometric parameters in the healthy population. A horizontal cross line inside the plot represents the achieved percentage considering all the eyes, and bars overpassing this line describe a higher probability of the formula failing in a particular percentile. The eyes with shorter AXLs also showed an increase of the PE (25% to 50%), in agreement with that shown in Figure 1B.

### 3.2. Standard Results

#### 3.2.1. Safety

No surgery complications were reported beyond 11 eyes with IFIS without affecting CDVA, 10 eyes with CDVA ≤ 0 logMAR and one eye with CDVA equal to 0.2 logMAR. Nine eyes presented PCO at six months, two of them requiring Nd:YAG. The cumulated number of eyes presenting PCO at 12 months was increased to 37 eyes, seven eyes requiring Nd:YAG. Only one eye lost two lines of CDVA at 12 months in comparison to the preoperative visit, mainly due to PCO, this being one of the previously described eyes requiring Nd:YAG.

#### 3.2.2. Efficacy and Predictability

**Figure 2:**
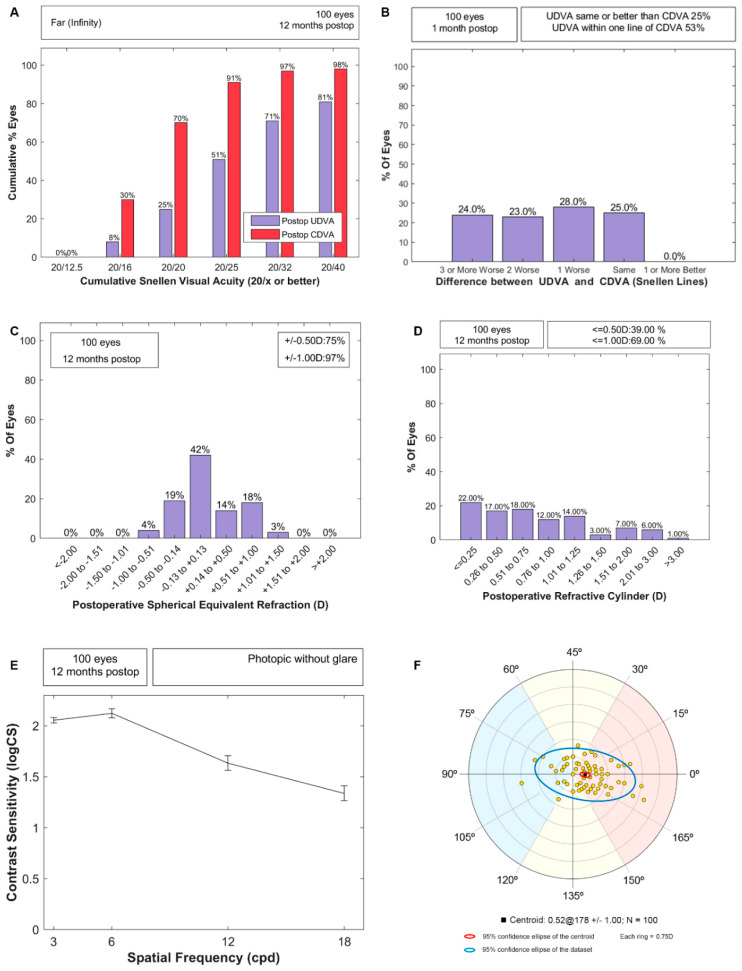
Standard outcomes at a 12-month visit for monocular vision. (**A**) Efficacy through postoperative uncorrected (UDVA) versus corrected (CDVA) postoperative visual acuities; (**B**) difference of lines between postoperative UDVA and CDVA; (**C**) spherical equivalent from subjective refraction; (**D**) refractive astigmatism distribution; (**E**) contrast sensitivity function with best distance correction; (**F**) Vector analysis of postoperative refractive astigmatism.

Figure 2A,B shows the standard efficacy plots; the median (IQR) for UDVA and CDVA were 0.1 (0.29) and 0 (0.12) logMAR, respectively. Figure 2C,D shows the spherical equivalent and residual refractive cylinder distributions, and the residual tendency of the cylinder is shown in Figure 2F. 61% and 31% of eyes resulted in a refractive astigmatism above 0.50 D and 1.00 D at 12 months, close to the 66% and 20% shown preoperatively by corneal astigmatism. The monocular contrast sensitivity function with the best distance correction is represented in Figure 2E. No significant association was found between the PE variation from one to 12 months and either the PCO manifestation (*p* = 0.68) or Nd:YAG (*p* = 0.09); therefore, the PE variation with a follow-up was not due to Nd:YAG.

## 4. Discussion

Standards for evaluating the PE from several formulas have been recently published without a uniform consensus about the amount of time after surgery that is required to measure the refractive error. While Hoffer and Savini recommended three months and at least two weeks to one month [7], Holladay et al. [6] were more conservative and suggested at least six months to achieve an effective lens position (ELP) stabilization. In our study, we evaluated the refractive error stabilization, obtaining a better agreement with Holladay et al. [6]. This does not mean that stabilization cannot be produced earlier than six months [17], but in some cases, as in our study, longer periods of time can be required. Both standard proposals agree that objective refraction should not be used to evaluate the PE [6,7]; however, there are still authors reporting the PE in terms of objective refraction [12,13,14]. Our results have shown that variations of SE can be detected with either objective or subjective refraction but that objective refraction results in more myopic residuals, something that can be less affected by open-field refractors [18]. Therefore, authors should avoid the use of objective refraction to assess the PE.

The hyperopic shift obtained in our study suggests a backward movement of the IOL after the first month. Koeppl et al. [19] reported a myopic shift due to the decrease of the distance between the anterior corneal vertex and the anterior intraocular lens surface, known as the actual lens position (ALP), during the first week after surgery, in agreement with Petternel et al. [20]. The latter also reported an increase of the ALP and a hyperopic shift from one week to three months, in agreement with the findings of our study. These shifts have been mainly explained by the transition between the optic and haptic design, with a higher movement in IOLs with sharp edges [20], by using single or multi-piece platforms [21], depending on the c-loop or four-point platforms [22] or on the area of connection between the haptic and the optic design [23]. The relationships between the AXL and IOL power with the hyperopic shift suggest that this effect should be particularly investigated in future studies, putting special emphasis on short eyes implanted with a high IOL power for which the ALP variation can produce a higher change in SE than IOLs with a lower power. Although conclusions could not be made through the descriptive analysis of PE for several biometric parameters, our results also suggest that eyes with longer LT and bigger WTW should be further explored in future studies, having not previously been described for the Barrett formula [15]. On the other hand, in our study, the percentages of eyes in ± 0.50 D and ± 1.00 D after refraction stabilization were 67% and 90%, respectively. This percentage could be improved with constant optimization, but this optimization should be conducted with 6-month refractions at least. After optimization, the Barrett accuracy will result in percentages above 79% and 94%, respectively, which were shown at one month and for which constant optimization was also required. This expected accuracy for the Barrett formula after optimization is consistent with the previously reported data in the literature for monofocal IOLs [24,25].

With regard to the standard results for this particular IOL, the Nd:YAG required rates were slightly higher than the expected ones for hydrophobic IOLs according to the results reported by the Royal College of Ophthalmologists in the 2021 audit [26]. 7% of eyes in our study required Nd:YAG, whereas for hydrophobic IOLs, 1% required it in this audit. Although we reported 37% of PCO at 12 months, this resulted in a decrease of CDVA of ≥2 lines in only six eyes from the total sample of 100. This is the reason why CDVA and CSF showed mean normal results, the latter in comparison with other studies using the same CSF test [27,28,29]. It is important to note that these CSF results could not be compared with those reported by other CSF tests, since the CC-100XP device usually overestimates the CSF in comparison with other tests such as the FACT, CSV-1000 or ClinicCSF [30].

Our study has some limitations to remark on. First, no measurements were taken for the ALP or pupil diameter during the follow-up visits. Thus, even though we have hypothesized that an ALP increase between one month and six months might explain the hyperopic shift, this should be demonstrated in future studies with the measurement of the real ALP. It is important to note that the intraocular lens induced a spherical aberration with the pupil diameter and intraocular lens power being unknown, and we cannot therefore hypothesize about their possible influence on the results. Furthermore, measurements were not obtained at three months, and we can say that the refractive error appears to be stabilized between one and six months but not at one month, at least for our sample and for this IOL. Other confounding factors, not measured in the current study, such as the capsulorhexis shape, might have had some influence on the hyperopic shift beyond the haptic design [31]. Second, the decision criteria for implanting the IOLs was limited by the possibilities offered to patients in accordance with the public health system. Due to this reason, a high percentage of eyes with a corneal astigmatism higher than 0.75 D were implanted with a spherical IOL instead of a toric IOL, which would lead to a better postoperative UDVA. This explains why only 53% of eyes obtained an UDVA with a maximum deviation of one line of visual acuity in comparison to CDVA. However, this second limitation has no influence on the PE assessment, which is evaluated through the spherical equivalent refraction.

## 5. Conclusions

In conclusion, our results suggest that some caution should be taken in studies reporting PE with refractive errors measured either objectively or at a 1-month follow-up, at least for this particular IOL model, which showed a hyperopic shift from one month to six months. Surgeons using this IOL should optimize its constant with a subjective refraction obtained at six months, even though a period of time between three and six months, not evaluated in this study, might also be valid. On the other hand, this is the first study to report standard results for this IOL model, which could be of special interest for surgeons who are considering to use this IOL.

## Figures and Tables

**Table 1:** Demographic characteristics of the sample.

Variable	Mean ± SD	Median [IQR]	Range [Min–Max]
Age	73 ± 7	74 (10)	52–90
Anterior Chamber Depth (mm)	3.25 ± 0.38	3.27 (0.49)	2.46–4.57
Axial Length (mm)	23.28 ± 0.93	23.29 (1.23)	21.19–25.73
Average Corneal Power (D)	43.91 ± 1.38	43.87 (1.90)	41.10–47.82
Corneal Astigmatism (D)	0.79 ± 0.59	0.69 (0.60)	0.02–3.74
Corneal diameter (mm)	11.97 ± 0.43	12 (0.58)	10.64–13.10
Lens Thickness (mm)	4.46 ± 0.45	4.5 (0.64)	3.31–5.57
Intraocular Lens Power (D)	22.48 ± 2.63	22.5 (3)	14–31
Target Refraction (D)	−0.19 ± 0.12	−0.17 (0.19)	−0.52–0.06

SD: Standard deviation; IQR: Interquartile range.

## Data Availability

The data that support the findings of this study are available from the author upon request.
